# Evaluation of Antidepressant Effect of Minocycline in Alcohol Abstinence-Induced Depression Model in Mice

**DOI:** 10.7759/cureus.28711

**Published:** 2022-09-02

**Authors:** Snehalata Gajbhiye, Arun Bhangre, Raakhi K Tripathi, Sharmila Jalgaonkar, Arun Shankar, Paresh G Koli

**Affiliations:** 1 Pharmacology and Therapeutics, Seth Gordhandas Sunderdas (GS) Medical College and King Edward Memorial (KEM) Hospital, Mumbai, IND

**Keywords:** animal research, psychiatry, alcoholic, ethyl alcohol, tetracycline

## Abstract

Introduction

Depression is one of the common comorbidities seen in chronic alcohol use disorder. Also, alcohol withdrawal induces depression and anxiety, which is associated with relapse in alcohol consumption. Minocycline, a tetracycline derivative, has shown an antidepressant effect in preclinical models. However, their effect on alcohol withdrawal-induced depression has not been studied. Therefore, the current study has been undertaken to evaluate the effect of minocycline on alcohol abstinence-induced depression models in mice.

Method

We conducted the study in two models. C57bl/6 mice were given a two-bottle choice (alcohol + water) for 28 days. During alcohol abstinence of 14 days, mice were treated with 10 mg/kg, 30 mg/kg, and 50 mg/kg of minocycline and were evaluated for behavioral changes using the forced swim test (FST) and tail suspension test (TST). A sucrose preference test was carried out where mice were exposed to binge alcohol drinking protocol for 12 days, where a two-bottle choice (alcohol or water) was given. This was followed by exposing the mice to a two-bottle choice paradigm (alcohol + sucrose) and they were divided into groups - no treatment group, vehicle-treated, minocycline 30 mg/kg or minocycline 50 mg/kg treated - and consumption of sucrose was assessed.

Result

In the forced swim test, a significant decrease in immobility time (p<0.05) was observed in the high-dose minocycline group (82.75±19.09) as compared to the vehicle control group (128.12±35.44). In the tail suspension test also, a significant decrease in immobility time (p<0.05) was seen in the high-dose minocycline group (83.75±18.61) as compared to the vehicle control group (122.25±18.51). The water and alcohol intake were comparable among all groups. In the sucrose preference test, it was found that the minocycline 50 mg/kg group had the highest sucrose preference (55%) followed by the minocycline 30 mg/kg group (50%) as compared to 42% in the vehicle control group. Significant reduction in brain-derived neurotrophic factor (BDNF) levels was seen with minocycline 50 mg/kg (p<0.05) and minocycline 30 mg/kg group (p<0.05) in BDNF levels when compared to the normal control group.

Conclusion

Minocycline in a higher dose (50 mg/kg) has shown an effect in alcohol withdrawal-induced depression in the abstinence-induced two-bottle choice model in mice. Both doses of minocycline have shown an effect in the sucrose preference test in the alcohol withdrawal-induced depression model.

## Introduction

Depression associated with alcohol use disorder is a widespread neuropsychiatric disorder worldwide. The extent of comorbidity between depression and alcohol use disorder was demonstrated by the results of several large epidemiological studies [1]. Alcohol use disorders (AUD) continue to be a concerning health issue worldwide [2]. Harmful alcohol use leads to 2.5 million deaths annually worldwide [2]. Individuals with alcohol abuse and/or dependence are typically at two to three times the risk for both the aggregated categories of anxiety (simple phobia, social phobia, agoraphobia, panic) and depressive disorders (major depression, dysthymia) [3]. Heavy drinking can lead to dependence, which in humans, is generally characterized by over 12-14 drinks per week [4]. Symptoms emerging after discontinuation of prolonged and excessive alcohol consumption are characteristic of alcohol dependence syndrome or alcohol use disorder [5].

The alcohol withdrawal syndrome can cause depression and other affective/mood disorders. Depression is characterized by a sad mood, anhedonia, feelings of worthlessness or guilt, suicidal ideation, changes in weight, appetite, sleep, and energy, as well as psychomotor and attention deficits. In humans, serotonin, dopamine, nor-adrenaline, and glutamate may play a large role in the pathophysiology of major depression disorder (MDD). Studies have shown that glutamate levels increase in the cerebrospinal fluid and increase in the brain [6]. Other than the monoamine hypothesis, it is postulated that improvement in depressive symptom severity is associated with a decrease in cerebral glutamate/glutamine levels [6]. The other symptom involved with alcohol withdrawal is anxiety [7]. Anxiety is frequently observed during and soon after detoxification [8].

Multiple neurotransmitters are involved in alcohol withdrawal syndrome like serotonin (5-HT), dopamine (DA), Nor-adrenaline, γ-Aminobutyric acid (GABA), and glutamine. Among all these serotonin, dopamine, nor-adrenaline and GABA are hypothesized to be involved in the pathophysiology of depression [9]. Withdrawal from alcohol causes a decrease in DA activity in the nucleus accumbens (NAC), as observed in rats' self-administered alcohol during withdrawal could rapidly reverse the deficit in DA and serotonin in the NAC [10]. It has been demonstrated that serotonin (5-HT) plays a facilitator role in DA release in the NAC while ethanol seems to directly stimulate the release of DA and 5-HT [11]. Chronic alcohol consumption enhances g-amino butyric acid (GABA) inhibitory action, while it blocks brain excitatory systems such as the glutamate N-methyl-D-aspartate (NMDA) receptor [12,13]. Thus, when alcohol is removed, GABA receptors are no longer stimulated while NMDA function is excessive. NMDA overactivity seems, among other pathways, to induce noradrenaline release, and may therefore contribute to the increased sympathetic activity observed during withdrawal [9].

There is a need for the development of drugs in this area as there is no approved drug for depression associated with alcohol use disorder. Thus, we tested minocycline for this condition. Minocycline is a broad-spectrum tetracycline antibiotic, with a long half-life of 12-18 h. It is the most lipid-soluble of the tetracycline antibiotics and it has the greatest penetration into the cerebrospinal fluid (CSF) and central nervous system (CNS) [14]. Minocycline has anti-inflammatory, antioxidant, anti-glutamatergic, and neuroprotective actions as a prototype of a multi-target antidepressant [15]. It has shown an antidepressant effect in preclinical studies [16]. Apart from its anti-glutamatergic effects, it increases DA and dihydroxyphenylacetic acid (DOPAC) levels in the amygdala. Minocycline is hypothesized to have the ability to inhibit microglial activation, a process that has deleterious effects on neurogenesis and neuronal survival, which would justify its potential effectiveness in the treatment of neuroinflammatory and/or neurodegenerative disorders [17]. The effect of the minocycline on alcohol withdrawal-induced depression had not been studied yet to the best of our knowledge. Therefore, the current study was undertaken to see the effect of minocycline on alcohol abstinence-induced depression in mice.

This article was previously presented as a conference poster at the 51st Indian Pharmacological Society Conference on December 06, 2019.

## Materials and methods

Permission from the Institutional Animal Ethics Committee (Seth GS Medical College and KEM Hospital, Mumbai) was taken before the commencement of the study. Animals randomly bred in the Advanced Centre for Treatment, Research, and Education in Cancer, Mumbai were procured and used. The study was conducted under the Committee for the Purpose of Control and Supervision of Experiments on Animals (CPCSEA) guidelines.

Part 1 - Evaluation of anti-depressant effect using forced swim test of minocycline on alcohol abstinence-induced depression model in mice

**Figure 1:**
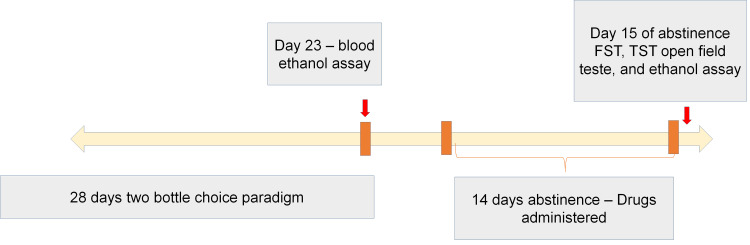
Flow of events in Part 1 FST: Forced swim test; TST: Tail suspension test.

C57BL/6j mice were divided into five groups (Table 1), with eight animals per group (n=8). Mice were weighed and handled daily for seven days before the procedure to facilitate adaptation to the laboratory. During the one-week period of acclimation and handling, water was the only fluid available. All fluids were presented in the home cage through 50 ml plastic centrifuge bottles with stainless steel sipper tubes. The model was initiated after a seven-day period of adaption. Alcohol-drinking mice were allowed to voluntarily consume alcohol (ethanol 10% v/v) or water for 28 days using a two-bottle drinking procedure. Water-only controls had access to two bottles containing water for 28 days. Animals were weighed and the fluid levels in the bottles were monitored to the nearest 0.5 ml at 24-hr intervals to determine daily fluid intake (ml and g/kg). The position (left-right) of the alcohol and water bottle was changed each day to control for side preferences. The two-bottle choice paradigm was carried out for 28 days (Figure 1) [18].

**Table 1:** Groups receiving treatment in part 1 of the study (using forced swim test in alcohol withdrawal-induced depression model)

Sr. no	Groups	Two bottles choice	Drug	No. of mice
1	Control	Water only		8
2	Vehicle control	Alcohol 10% v/v and water	Vehicle	8
3	Test group 1		Minocycline 10 mg/kg orally	8
4	Test group 2		Minocycline 30 mg/kg orally	8
5	Test group 3		Minocycline 50 mg/kg orally	8

On day 23 of alcohol drinking, retro-orbital blood was collected and analyzed for blood alcohol levels using the Abcam ethanol assay kit. On the 29th day, the alcohol solution-containing bottles were removed from the cages of alcohol-drinking mice, and one water bottle was removed from the cages of controls; thus, during abstinence, only one bottle was in each cage. This was taken as the 1st day of abstinence.

Animals were randomly assigned to study groups (Table 1). It was assured that mean ethanol intake is balanced across groups. Alcohol-drinking mice were then treated with minocycline (10 mg/kg, 30 mg/kg, and 50 mg/kg) [19] or vehicle during a 14-day abstinence period (Table 1). Water-only control mice did not receive any treatment. All mice were evaluated for locomotor activities in an open field test, followed by the tail suspension test and forced swim test (FST) for immobility time one day after the final dose of minocycline, to examine the antidepressant effect of the treatment.

Forced swim test

The rationale for the test is based on the assumption that when placing an animal in a container filled with water, it will first make efforts to escape but eventually will exhibit immobility that may be considered to reflect a measure of behavioral despair.

For the forced swim test, mice were placed individually in a cylindrical Plexiglas tank (25 cm high x 12 cm diameter), filled with 15 cm of water. The time spent immobile during the 6-min FST was recorded. Mice were counted as immobile when they remained floating passively in the water in a slightly hunched but upright position, their nose just above the water with minimal movement of their limbs. Mice were removed from the cylinder immediately after the test, dried with paper towels, and kept under a heating lamp until completely dry before returning to their home cages.

Tail suspension test

The rationale for the test is based on the assumption that when placing an animal at the edge of a table from a height, it will first make efforts to escape but eventually will exhibit immobility that may be considered to reflect a measure of behavioral despair.

Sticking tape over the tail of mice with its ends fixed to the edge of a table at a height of 58cm was applied. Each mouse was left in this position for 5 minutes. Observation of the time for which the mice remain immobile was recorded.

Open field test

It is a sensorimotor test to determine gross locomotor activity and exploration habits in mice. If a drug affects locomotion, then it has a propensity to show false positive on the model of depression. To rule out the effect on the model is not due to its effect on locomotion, an open field test is performed.

All the alcohol-dependent mice were subjected to an open field test (OFT). OFT apparatus comprises floor 60x60 cm, wall height 25 cm in dimension. The floor and wall were black, ceiling was open, floor was divided into nine equal squares. The apparatus was coupled with a video device positioned above the apparatus so that each trial can be recorded for later analysis, using a video camcorder. The apparatus was kept for the test in a sound attenuated, dark room with minimal background lighting. The mice were placed either in the center or against one of the retaining walls. Mice were allowed to explore the apparatus for 15 minutes. The variable recorded was the total distance traveled in the open field test.

Variables recorded were blood alcohol level (ethanol assay kit), daily alcohol intake (ml and gm/kg), immobility time in forced swim test and tail suspension test, and total distance traveled on open field test.

Part 2 - To study the antidepressant effect of minocycline using sucrose preference test on alcohol abstinence-induced depression model in mice

**Figure 2:**
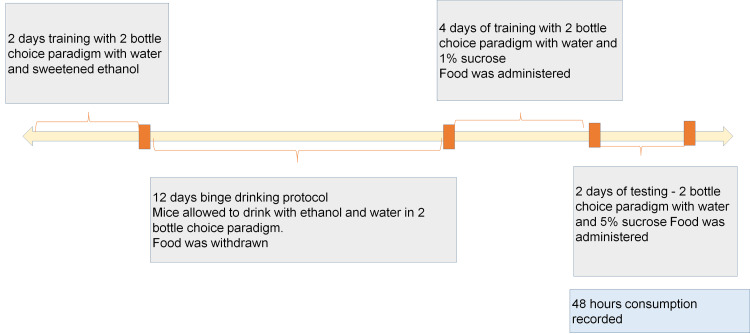
Flow of events in Part 2

Mice were kept at 14:10 reverse light-dark cycle. Mice were given training for the first two days, wherein two daily sessions of 30-minute duration which was at least 1 hour apart were given. At this time, mice were exposed to two-bottle choice paradigm 4 hours into the dark cycle. Food was removed and two bottles were placed on the cage lid: one containing water and the other containing sweetened ethanol (10% w/v) prepared with 95% ethyl alcohol and tap water + 3% glucose and 0.125% saccharin. The position of the bottles was alternated daily to avoid possible side preferences. For the control group, two bottles containing water were placed (Figure 2) [20].

After mice acquired the behavior during training, an experimental binge drinking protocol was started the following day. During the experimental period, bottles with ethanol and water were attached to the cage lid starting 4 hours into the dark cycle, and mice were allowed to drink for 30 minutes. The position of the bottles was alternated daily to avoid possible side preferences. During this time, food was removed. The binge drinking protocol was conducted for 12 consecutive days. All bottles were weighed immediately before and after the 30-min drinking sessions. Differences in bottle weights were converted to volume intakes by accounting for the ethanol solution density (weight in grams/0.9868). At the end of the binge alcohol protocol, the animals' blood samples were taken to measure blood alcohol levels using an ethanol assay kit.

To assess depression-like symptoms, a sucrose preference test was carried out after the last drinking session. For this test, mice were acclimated to a two-bottle choice of drinking water and 1% sucrose solution for four days as a part of training. The training was followed by two days of testing. On the day of testing, two pre-weighted bottles of 5% sucrose solution and tap water were presented. To prevent side preferences in drinking behavior, the position of the bottles was switched after 24 hours of testing. No food or water deprivation was applied before or during the test.

All animals were administered test drugs during four days of training (Table 2) and evaluation of sucrose preference was tested. Liquid consumption from each bottle corrected by body weight was used to calculate sucrose solution intake, water intake, and total consumption by the end of the 48 hours. Sucrose preference was calculated using the following equation: Sucrose preference (%) = sucrose intake/ (sucrose intake + water intake) × 100.

**Table 2:** Groups receiving treatment in part 2 of the study (using sucrose preference test in alcohol withdrawal-induced depression model)

Sr.no	Groups	Two bottles choice	Drug	No. of mice
1	Control	Water only		8
2	Vehicle control	Alcohol 10% v/v and water (from day 1 to day 14). Alcohol 10% v/v and 1% sucrose (days 15 to 18). Alcohol 10% v/v and 5% sucrose (on day 19 and day 20)	Vehicle	8
3	Test group 1		Minocycline 30 mg/kg	8
4	Test group 2		Minocycline 50 mg/kg	8

After 48 hours of recording, sucrose consumption animals were sacrificed, and their brain (hippocampus) were preserved and assessed for brain-derived neurotrophic factor (BDNF) levels using the BDNF ELISA kit. Variables recorded were daily alcohol intake (ml and g/kg), blood alcohol level (ethanol assay kit), percentage of sucrose preference (%) calculated by the formula: sucrose intake/ (sucrose intake + water intake) × 100, number of line crosses, distance traveled, frequency of rearing in open field test and detecting BDNF levels using BDNF ELISA kit.

Statistical analysis

All continuous variables were expressed as Mean ± SD. The sucrose preference is expressed in percentage. The between-group comparison was done by one-way ANOVA followed by the post hoc Tukey's test. All data were entered in a Microsoft excel sheet and analysis was done using SPSS version 25 (IBM Corp., Armonk, NY).

## Results

The results are presented as parts 1 and 2.

Part 1 - Anti-depressant effect of minocycline using the forced swim test of minocycline on alcohol abstinence-induced depression model

Blood Alcohol Level

Blood alcohol level estimation using an ELISA kit was done on day 23 of the two-bottle choice paradigm. There was a significant increase in blood alcohol levels in all the alcohol-exposed groups as compared to water only group as shown in Table 3. The rats were further allocated to different treatment groups based on their alcohol consumption such that all the groups had rats with comparable alcohol drinking.

**Table 3:** Blood alcohol levels in groups of part 1 (n=8) Data expressed in Mean ± SD, as compared to the normal control group after applying ANOVA followed by post hoc Tukey's test.

Groups	Blood alcohol level in mg/dl	P-value
Normal control	2.03 ± 0.77	
Vehicle control	36.31 ± 4.50*	<0.001
Minocycline 10 mg/kg	34.2 ± 1.40	<0.001
Minocycline 30 mg/kg	35 ± 1.1	<0.001
Minocycline 50 mg/kg	34.39 ± 0.8	<0.001

Alcohol Drinking

The alcohol drinking was assessed daily and the mean alcohol intake per day for 28 days in various groups is depicted in Table 4. The alcohol intake across groups was comparable.

**Table 4:** Alcohol intake in treatment groups (n=8) Data expressed in Mean ± SD, as compared to the normal control group after applying ANOVA followed by post hoc Tukey's test.

Groups (n=8)	Alcohol intake in gm/kg/day	P-value
Vehicle control	5.21 ± 0.88	_
Minocycline 10 mg/kg	5.29 ± 1.35	0.99
Minocycline 30 mg/kg	5.25 ± 0.94	0.99
Minocycline 50 mg/kg	5.25 ± 1.3	0.99

Immobility Time in Forced Swim Test and Tail Suspension Test

**Figure 3:**
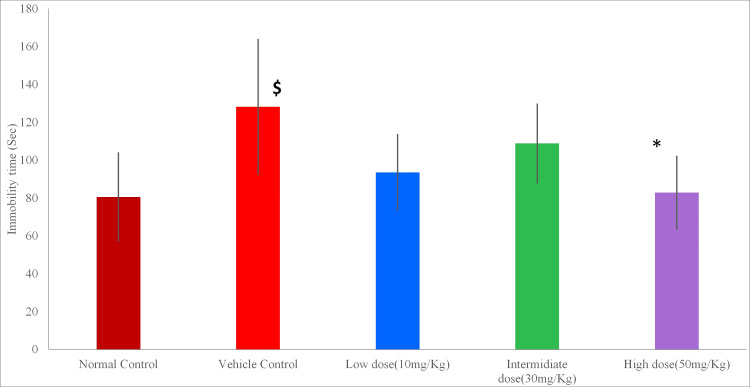
Immobility time in forced swim test Data expressed in Mean ± SD; ^$^p<0.05 vs normal control, *p<0.05 vs Vehicle control; one-way ANOVA test followed by post hoc Tukey's test

Immobility time in forced swim test. There was a significant increase in immobility time in the vehicle control group as compared to the normal control group (p=0.004), showing the development of depression in alcohol-exposed rats. There was a significant decrease in immobility time in the high-dose minocycline group (50 mg/kg/day) as compared to the vehicle control group (p=0.006). The minocycline 10 mg/kg (p=0.55) and 30 mg/kg (p=0.516) did not show statistical significance. Result values are represented graphically in Figure 3.

**Figure 4:**
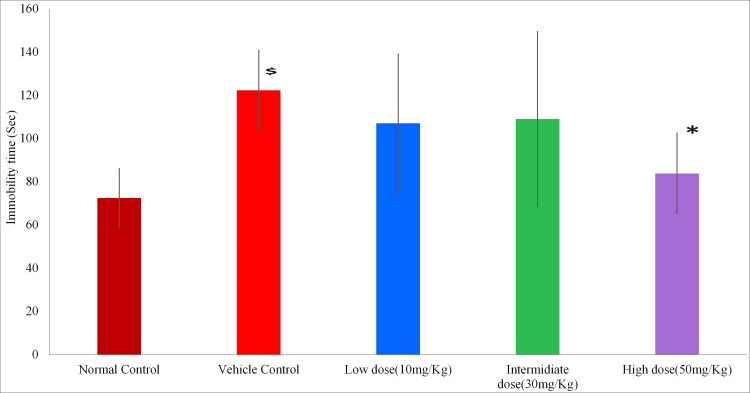
Immobility time in tail suspension test Data expressed in Mean ± SD; ^$^p<0.05 vs normal control, *p<0.05 vs vehicle control; one-way ANOVA test followed by post hoc Tukey's test

Immobility time in tail suspension test. In the tail suspension test, there was a significant increase in immobility time in the vehicle control group as compared to the normal control group (p=0.005), indicating the development of depression in alcohol-exposed rats. There was a significant decrease in immobility time in the high-dose minocycline group (50 mg/kg/day) as compared with the vehicle control group showing a protective effect of minocycline in depression (p=0.048). The minocycline 10 mg/kg (p=0.781) and 30 mg/kg (p=0.855) doses could not achieve statistical significance as compared to the vehicle control group. Values are represented graphically in Figure 4.

Open Field Test

We evaluated the behavior of animals in the open field test for any locomotor disturbance. In this test, the total distance traveled (in cm) by animals in the open field is recorded using an overhead video tracking system with software (Maze Master). In the open field test, there was no significant change in the total distance traveled across the groups. Values are presented in Table 5.

**Table 5:** Total distance traveled in open field test Data expressed in Mean ± SD; groups vs vehicle control; one-way ANOVA followed by post hoc Tukey's test applied; n=8

Groups (n=8)	Total distance traveled in cm	P-value
Normal control	5741.15 ± 1155.24	0.462
Vehicle control	4845.61 ± 1157.80	_
Minocycline 10 mg/kg	5076.82 ± 745.27	0.992
Minocycline 30 mg/kg	5201.04 ± 1172.97	0.963
Minocycline 50 mg/kg	5061.59 ± 1052.92	0.994

Part 2 - Antidepressant effect of minocycline using sucrose preference test on alcohol abstinence-induced depression model

Daily Alcohol Intake (g/kg)

The alcohol intake in g/kg on day 14 of the experiment (i.e., the last day of the experimental binge protocol of 12 days) was compared between the groups. It was seen that there was no statistically significant difference in alcohol intake between vehicle control, minocycline 30 mg/kg, and minocycline 50 mg/kg groups. The normal control group is not depicted as the animals were not exposed to alcohol. Values are presented in Table 6.

**Table 6:** Alcohol intake in treatment groups Data expressed in Mean ± SD; groups vs vehicle control; one-way ANOVA followed by post hoc Tukey's test applied; n=8

Groups (n=8)	Alcohol intake in g/kg	P-value
Normal control	-	_
Vehicle control	61.80 ± 22.26	_
Minocycline 30 mg/kg	65.28 ± 19.27	0.92
Minocycline 50 mg/kg	58.68 ± 12.93	0.93

Blood Alcohol Level (Ethanol Assay Kit)

Blood alcohol level estimation using an ELISA kit was done after the experimental binge drinking protocol on day 14 of the two-bottle choice paradigm. There was a significant increase in blood alcohol level in vehicle control, minocycline 30 mg/kg, and minocycline 50 mg/kg group as compared to the normal control group (water only group) with a p-value of <0.01. Values are presented in Table 7.

**Table 7:** Blood alcohol level in treatment groups Data expressed in Mean ± SD; One-way ANOVA and post hoc Tukey's test; n=8

Groups (n=8)	Blood alcohol level in mg/dl	P-value
Normal control	1.67 ± 0.58	
Vehicle control	34.82 ± 3.28	<0.001
Minocycline 30 mg/kg	36.30 ± 0.86	<0.001
Minocycline 50 mg/kg	35.83 ± 0.36	<0.001

Percentage of Sucrose Preference (%)

**Figure 5:**
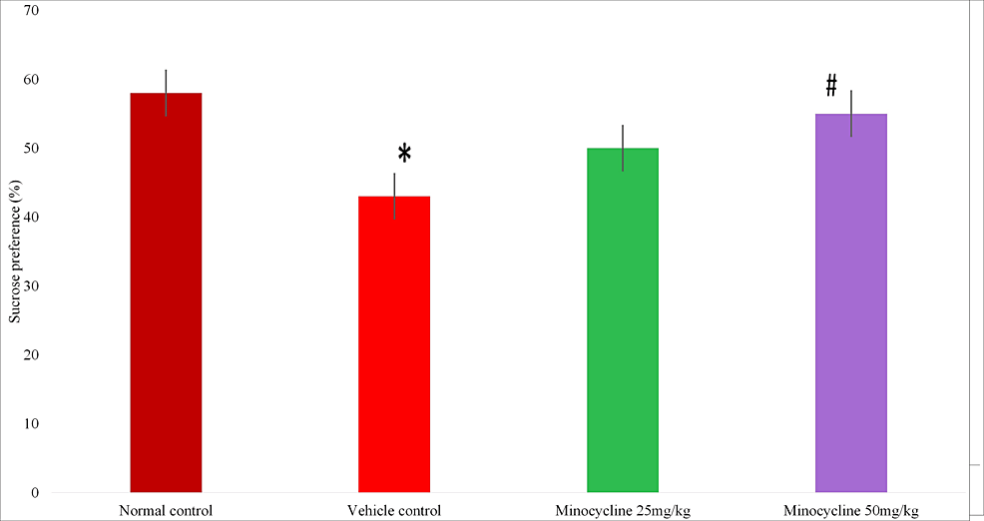
Sucrose preference (%) Data expressed in percentage; * p < 0.05 vs normal control, #p < 0.05 vs vehicle control; One-way ANOVA and post hoc Tukey's test; n=8

This was calculated by the following formula, i.e., sucrose intake/(sucrose intake + water intake) × 100. There was a significant increase (Figure 5) in sucrose preference in the normal control group when compared to the vehicle control group (p<0.05) and in the vehicle control group when compared to the minocycline 50 mg/kg group (with p<0.05).

BDNF Levels

**Figure 6:**
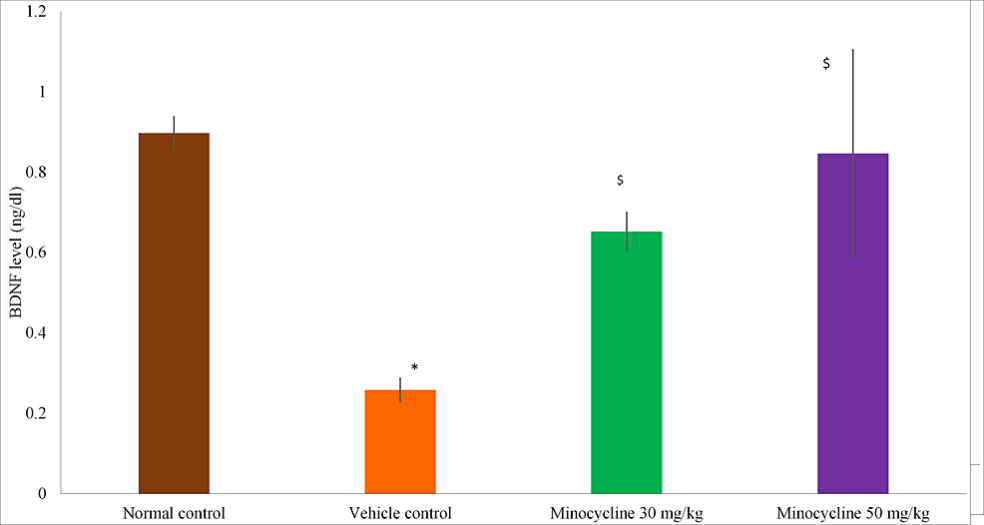
Brain-derived neurotrophic factor (BDNF) levels in different groups Data expressed in Mean ± SD; *p<0.05 vs Normal control; ^$^p<0.05 vs Vehicle control; One-way ANOVA and post hoc Tukey's test; n=8

The animals were sacrificed on day 21 and hippocampus of the brain was dissected and BDNF levels were measured. The vehicle control group showed a significant reduction in BDNF levels as compared to the normal control group (p<0.05). There was a statistically significant increase in BDNF levels in test groups - minocycline 30 mg/kg group and minocycline 50 mg/kg as compared to the vehicle control group (p<0.05). The group receiving minocycline 50 mg/kg did not show significance as compared to the normal control group (p=0.8703) restoring BDNF levels to the normal range. Values are presented in Figure 6.

## Discussion

We propose that abstinence from chronic alcohol drinking leads to depression-like behavior and treatment with minocycline at the dose of 50 mg/kg/day in the abstinence phase leads to a reduction in depression-like behavior in two different preclinical models. The study also shows a rise in BDNF levels, which is a marker for neurogenesis and neuronal plasticity in treated groups. Although low dose (10 mg/kg/day) and intermediate dose (30 mg/day/kg) also decreased immobility time and increased sucrose consumption, but they were not statistically significant. Studies have used different doses of minocycline while studying the antidepressant effects in various models of depression. The dose range of minocycline used in animal studies ranges from 5 mg/kg [21] to 160 mg/kg [22]. A study conducted by Mahmoud et al. has shown that minocycline in the dose of 10 mg/kg showed an antidepressant effect in T. gondii-induced anhedonia [23]. Also, another study by Singh and Goel studied the minocycline doses of 10, 20, and 40 mg/kg in depression associated with epilepsy. This study also showed that lower doses took a while to show antidepressant effects [24]. However, in this study, we found a significant difference with the dose of 50 mg/kg. We examined the behavior of mice for general locomotion and found that minocycline does not have any effect on the general locomotory behavior. This finding is supported by other studies [22,25].

Alcohol-induced depressive disorder refers to a depression-like syndrome that occurs during and at the time of alcohol intoxication or abstinence and is usually associated with distress and impairment [26]. Grant et al. have shown that persons with AUD are 2.3 times more likely to also have a major depressive disorder and they are 1.7 times more likely to have dysthymia [27]. There are also twin studies showing that genetic factors influence susceptibility to developing major depressive disorder and alcohol consumption [28]. In this study, we chose the voluntary alcohol drinking model to evaluate depression-related behavior in the alcohol abstinence phase because this model has more clinical relevance than forced alcohol consumption as it replicates the alcohol abstinence-induced depression in humans [29]. The models of depression chosen for the present study were such that one is a model of behavior despair and the other highlights anhedonia, thus, indicating two different symptomatology of depression. We also carried out the blood alcohol analysis to ensure that all rats randomized to different treatment arms have been matched for alcohol intake.

Minocycline, a tetracycline antibiotic, has potent anti-inflammatory and neuroprotective effects. Minocycline has been shown to have an effect on alcohol use disorder. In a study conducted by Karelina et al., it was found that minocycline when administered to traumatic brain injured mice reduced alcohol consumption and the probable mechanism proposed was inhibition of microglial activation [30]. Also, in another study conducted on alcohol-deprived rats, minocycline reduced the chance of developing alcohol relapse by reducing cytokine levels [18]. In line with our study, minocycline has shown an effect on depression in different models. A study by Camargos et al. evaluated the effect of minocycline on transient global cerebral ischemia in C57BL/6 mice and the study showed the antidepressant and anxiolytic effect of minocycline owing to its neuroprotective effect after brain ischemia in mice [31]. A study by Du et al. evaluated the effect of minocycline on transient global cerebral ischemia in an animal model and showed that minocycline exerts an anti-depressant effect by inhibiting microglia activation, promoting oligodendrocyte progenitor cells (OPCs) maturation and remyelination [32]. A study by Zhang et al. demonstrated that minocycline improves anxiety-related behaviors in female Sprague-Dawley rats subjected to chronic unpredictable mild stress by altering hippocampal neuroinflammation, GABA, and serum cholesterol levels [33]. The study shows that minocycline has antidepressant-like effects on rats.

Depression downregulates hippocampal BDNF showing a neuronal loss. Similar to our findings, a study conducted by Briones and Woods showed chronic binge drinking in male Sprague-Dawley rats led to the development of a depressive phenotype, decreased survival, and neuronal differentiation of neural progenitor cells in the hippocampus, and decreased BDNF effect during the withdrawal period [34]. Thus, showing that alcohol withdrawal results in a depressive phenotype and associated decrease in BDNF. Also augmenting BDNF actions using tyrosine kinase B agonist restored neurogenesis and abolished the alcohol-induced anhedonia and despair behaviors seen during the abstinence period. In the current study, the mice in the vehicle control group showed a highly significant reduction in BDNF levels when compared to the normal control group, while mice in the minocycline 50 mg/kg group showed improved levels of BDNF showing restored neurogenesis.

## Conclusions

This study evaluated the anti-depressant effect of minocycline on alcohol abstinence-induced depression models in mice and found that minocycline in higher dose (50 mg/kg) showed antidepressant effect and lowered BDNF levels, showing restored neurogenesis. The anti-depressant effect of minocycline in the alcohol abstinence-induced depression model could be further evaluated using histological parameters, as this was not assessed in our study. Further studies exploring the anti-depressant mechanisms of minocycline at a molecular level should be carried out.
